# The commensal consortium of the gut microbiome is associated with favorable responses to anti-programmed death protein 1 (PD-1) therapy in thoracic neoplasms

**DOI:** 10.20892/j.issn.2095-3941.2020.0450

**Published:** 2021-05-07

**Authors:** Huihui Yin, Lu Yang, Gongxin Peng, Ke Yang, Yuling Mi, Xingsheng Hu, Xuezhi Hao, Yuchen Jiao, Xiaobing Wang, Yan Wang

**Affiliations:** 1 State Key Laboratory of Molecular Oncology, National Cancer Center/National Clinical Research Center for Cancer/Cancer Hospital, Chinese Academy of Medical Sciences and Peking Union Medical College, Beijing 100021, China; 2 Department of Medical Oncology, National Cancer Center/National Clinical Research Center for Cancer/Cancer Hospital, Chinese Academy of Medical Sciences and Peking Union Medical College, Beijing 100021, China; 3 Center for Bioinformatics, Institute of Basic Medical Sciences, Chinese Academy of Medical Sciences & School of Basic Medicine, Peking Union Medical College, Beijing 100021, China; 4 Department of Medical Oncology, Cancer Hospital of Huanxing Chaoyang District Beijing, Beijing 100122, China; 5 Department of Medical Oncology, Chaoyang Sanhuan Cancer Hospital, Beijing 100021, China

**Keywords:** Gut microbiota, commensal microbes, anti-PD-1 immunotherapy, thoracic neoplasms

## Abstract

**Objective:**

Immune checkpoint inhibitors have revolutionized cancer therapy for multiple types of solid tumors, but as expected, a large percentage of patients do not show durable responses. Biomarkers that can predict clinical responses to immunotherapies at diagnosis are therefore urgently needed. Herein, we determined the associations between baseline gut commensal microbes and the clinical treatment efficiencies of patients with thoracic neoplasms during anti-programmed death protein 1 (PD-1) therapy.

**Methods:**

Forty-two patients with advanced thoracic carcinoma who received anti-PD-1 treatment were enrolled in the study. Baseline and time-serial stool samples were analyzed using 16S ribosomal RNA gene sequencing. Tumor responses, patient progression-free survival, and overall survival were used to measure clinical outcomes.

**Results:**

The diversities of the baseline gut microbiota were similar between responders (*n* = 23) and nonresponders (*n* = 19). The relative abundances of the *Akkermansiaceae*, *Enterococcaceae*, *Enterobacteriaceae*, *Carnobacteriaceae* and *Clostridiales Family XI* bacterial families were significantly higher in the responder group. These 5 bacterial families acted as a commensal consortium and better stratified patients according to clinical responses (*P* = 0.014). Patients with a higher abundance of commensal microbes had prolonged PFS (*P* = 0.00016). Using multivariable analysis, the abundance of the commensal consortium was identified as an independent predictor of anti-PD-1 immunotherapy in thoracic neoplasms (hazard ratio: 0.17; 95% confidence interval: 0.05–0.55; *P* = 0.003).

**Conclusions:**

Baseline gut microbiota may have a critical impact on anti-PD-1 treatment in thoracic neoplasms. The abundance of gut commensal microbes at diagnosis might be useful for the early prediction of anti-PD-1 immunotherapy responses.

## Introduction

Increasing evidence has suggested that avoiding immune destruction during the pathogenesis of cancer is an additional hallmark of cancer^1^. Key immune evasive pathways, including the CD28/cytotoxic T-lymphocyte antigen 4 axis and the programmed death-ligand 1 (PD-L1)/PD-1 axis, which are known as immune checkpoint inhibitors (ICIs), are therefore promising therapeutic targets for drug development^2–5^. ICIs currently approved by the U.S. Food and Drug Administration for non-small cell lung cancer (NSCLC) include atezolizumab, nivolumab, and pembrolizumab^4,6,7^. Furthermore, the success of immunotherapy for NSCLC patients has led to similar benefits for patients with other rare thoracic malignancies, such as thymic epithelial tumors, mesothelioma, and small cell lung cancer (SCLC)^8–10^.

Due to the complexity of the immune system, immunotherapeutic biomarkers are fundamentally different from targeted therapy biomarkers. PD-L1 expression on cancer cells has always been a research focus^11^. In the KEYNOTE-024 trial involving patients with treatment-naïve advanced NSCLC with high PD-L1 expression on the surface of tumor cells and wild-type *EGFR* and *ALK*, pembrolizumab significantly improved progression-free survival (PFS), overall survival (OS), and the objective response rate (ORR)^12^. Unlike first-line treatment, PD-L1 status alone is not sufficient to ensure a response to ICI treatment using second-line therapy. The CheckMate 057 study showed that prolonged OS with nivolumab treatment was correlated with higher levels of tumor PD-L1 expression, but treatment efficacy was also reported in patients with less than 1% PD-L1 expressions^13^. In addition to tumor PD-L1 expression, tumor mutational burden, tumor lymphocyte infiltrate, peripheral blood biomarkers, and the gut microbiota are emerging biomarkers for checkpoint inhibitor-based immunotherapy^14–18^.

The gut microbiota, which is composed of 10^13^–10^14^ microorganisms, can be considered an endogenous factor that continuously influences daily life^19^. Animal models for microbiota studies have shown that it has an important effect on host physiology, including on the regulation and remodeling of immune responses^20–22^. Multiple studies have shown that gut microbes profoundly influence cancer immunotherapy^23–26^. Fecal DNA sequencing prior to ICI treatment identified a relationship between the gut microbiome compositions and therapeutic responses in NSCLC, renal cell carcinoma, and melanoma^17,27,28^. The aim of the present study was therefore to provide a clear understanding of the predictive potential of the gut microbiome prior to ICI therapy, by quantitating the relative percentages of putatively identified “beneficial” bacteria.

## Materials and methods

### Patients

**Figure 1:**
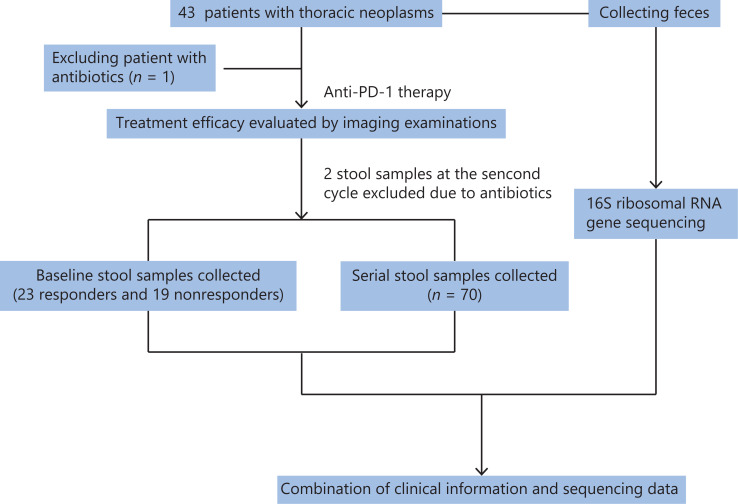
Flow chart of this study.

This retrospective study, from January 2018 to July 2019, included patients with advanced thoracic carcinoma at the National Cancer Center/National Clinical Research Center for Cancer/Cancer Hospital, Chinese Academy of Medical Sciences and Peking Union Medical College. The enrolled patients were diagnosed with stage IV thoracic carcinomas and initially received immune monotherapy. The exclusion criteria were patients receiving antibiotics (ATBs) at the initiation of immunotherapy. The flow chart for this study is shown in **Figure 1**. This study was approved by the ethics committee of the National Cancer Center/National Clinical Research Center for Cancer/Cancer Hospital, Chinese Academy of Medical Sciences and Peking Union Medical College.

Baseline characteristics were collected from medical records (**Table 1**). In general, treatment efficacy evaluation was performed by imaging examinations every 2 or 3 cycles. Patients receiving single-agent nivolumab were assessed every 3 cycles, and patients receiving other anti-PD-1 inhibitors were assessed every 2 cycles. Therapeutic response was evaluated as complete response (CR), partial response (PR), stable disease (SD), and progressive disease (PD) according to the Response Evaluation Criteria in Solid Tumors, version 1.1. Responders (R, *n* = 23) were defined as those with CR, PR, or SD. Nonresponders (NR, *n* = 19) were defined as those with PD. The ORR was defined as the percentage of patients experiencing an objective response (CR or PR) as the best response to anti-PD-1 therapy, while disease control rate (DCR) was categorized as the percentage of CR, PR, or SD. PFS was defined as the period from the initiation of anti-PD-1 antibody treatment to the date of disease progression. OS was defined as the time from anti-PD-1 therapy initiation to death. The last follow-up was April 27, 2020.

**Table 1:** Baseline characteristics of patients with thoracic neoplasms

Characteristics	Total (*n* = 42)	NR (*n* = 19)	R (*n* = 23)	*P*
Age, years				0.769
<65	30 (71.4%)	14 (73.7%)	16 (69.6%)	
≥65	12 (28.6%)	5 (26.3%)	7 (30.4%)	
Gender				1
Female	10 (23.8%)	5 (26.3%)	5 (21.7%)	
Male	32 (76.2%)	14 (73.7%)	18 (78.3%)	
ECOG PS				1
0	1 (2.4%)	0 (0.0%)	1 (4.3%)	
1	17 (40.5%)	8 (42.1%)	9 (39.1%)	
2	24 (57.1%)	11 (57.9%)	13 (56.5%)	
Smoking status				0.453
Nonsmoker	13 (31.0%)	7 (36.8%)	6 (26.1%)	
Smoker	29 (69.0%)	12 (63.2%)	17 (73.9%)	
Histology				0.087
Lung adenocarcinoma	15 (35.7%)	9 (47.4%)	6 (26.1%)	
Lung squamous carcinoma	23 (54.8%)	7 (36.8%)	16 (69.6%)	
Other^†^	4 (9.5%)	3 (15.8%)	1 (4.3%)	
Mutation status				0.122
*EGFR*	5 (11.9%)	4 (21.1%)	1 (4.3%)	
*ALK*	1 (2.4%)	1 (5.3%)	0 (0.0%)	
*KRAS*	1 (2.4%)	0 (0.0%)	1 (4.3%)	
WT/unknown	35 (83.3%)	14 (73.7%)	21 (91.3%)	
Metastasis sites				0.125
<2	14 (33.3%)	4 (21.1%)	10 (43.5%)	
≥2	28 (66.7%)	15 (78.9%)	13 (56.5%)	
Number of prior systemic regimens				0.002*
<3	30 (71.4%)	9 (47.4%)	21 (91.3%)	
≥3	12 (28.6%)	10 (52.6%)	2 (8.7%)	
Previous systemic therapy				0.808
Platinum-based therapy	36 (85.7%)	16 (84.2%)	20 (87.0%)	
Other systemic therapy	5 (11.9%)	2 (10.5%)	3 (13.0%)	
Unknown	1 (2.4%)	1 (5.3%)	0 (0.0%)	
Prior radiotherapy				0.525
No	15 (35.7%)	8 (42.1%)	7 (30.4%)	
Yes	27 (64.3%)	11 (57.9%)	16 (69.6%)	
Usage of ATB				1
No	40 (95.2%)	18 (94.7%)	22 (95.7%)	
Yes	2 (4.8%)	1 (5.3%)	1 (4.3%)	

*P < 0.05 was considered significant. ^†^Other included 1 SCLC, 1 NSCLC, 1 thymic squamous carcinoma, and 1 large cell neuroendocrine carcinoma. ATB: antibiotics.

### Sample collection and DNA extraction

Fresh feces were collected at the pretreatment visit and continuously collected before each cycle of infusion (**Supplementary Figure S1**). A collection kit including protectant medium was given to patients. Samples were collected by patients and frozen at −80 °C. Total fecal DNA extraction was conducted according to the noncommercial protocol Q recommended by the International Human Microbiome Consortium (http://www.human-microbiome.org/). The DNA concentration was determined using a Qubit^™^ dsDNA HS Assay Kit (Thermo Fisher Scientific, Waltham, MA, USA). DNA integrity was evaluated by 1% agarose gel electrophoresis. Samples with sterile water served as negative controls.

### PCR amplification and Illumina sequencing

PCR amplification of the V4 variable region was performed using 515F (5′-GTGYCAGCMGCCGCGGTAA-3′) and 806R (5′-GGACTACNVGGGTWTCTAAT-3′) primers. PCR products were purified using AMPure XP beads (Beckman Coulter, Brea, CA, USA). Purified amplified fragments were then amplified by 8 cycles of PCR using a KAPA HiFi HotStart Ready Mix (2×) (Roche, Basel, Switzerland) and Illumina adaptor-specific primers (Illumina, San Diego, CA, USA). Indexed libraries were then purified with AMPure XP beads (Beckmann Coulter) and sequenced on an Illumina HiSeq platform (Illumina). Details on the 16S ribosomal RNA gene amplicon preparation protocol with Illumina were previously described (https://www.illumina.com/).

### Sequence analysis

Sequencing data analysis was performed using QIIME version 1 and was grouped into operational taxonomic units (OTUs) against the SILVA (version 132) database at 97% similarity^29,30^. Rarefaction curve analysis, alpha diversity, and beta diversity of the gut microbiota were assessed by Microbiome Analyst packages^31^. The Chao1, Shannon, and Simpson indices were calculated to measure the richness and evenness of OTUs within a sample^31^. Beta diversity analysis between samples was assessed using the Bray-Curtis distance and visualized by principal coordinate analysis (PCoA) plots^31^. The cumulative-sum scaling method was used to determine the microbiome composition from phylum to genus levels^32^. Heat map visualization of gut microbiota was performed at the family level, and was clustered using hierarchical clustering with Euclidean distance.

### Statistical analysis

Statistical analyses were conducted with SPSS statistical software for Windows, version 22.0 (IBM, Armonk, NY, USA) and Prism 6.0 (GraphPad, San Diego, CA, USA). The Chao1, Shannon, and Simpson indices were used to compare the differences between samples, and a nonparametric Mann-Whitney test or Kruskal-Wallis test was used to compare differences in the relative abundance of taxa. Receiver operating characteristic (ROC) curves were used to calculate the cut-off value of 5 bacterial families and the commensal consortium. The Fisher test was performed to determine the correlation between the gut microbiota and response rate. Survival curves were estimated using the Kaplan-Meier method (log-rank test). Univariate and multivariate analyses were conducted using the Cox regression model. All reported tests were two-tailed and considered significant at *P* < 0.05.

## Results

### Patient characteristics

A total of 42 patients were enrolled in this study (**Table 1**). Thirty (71.4%) of the 42 patients were younger than 65-years-old at initial diagnosis, 32 (76.2%) of the 42 patients were male, and 29 patients (69.0%) were smokers. The performance status ranged from 0 to 2, with 57.1% of patients having a performance status equal to 2 prior to ICI initiation. Of these patients, 23 (54.8%) had lung squamous carcinomas, 15 had lung adenocarcinomas, 1 had a SCLC, 1 had a NSCLC, 1 had a thymic squamous carcinoma, and 1 had a large cell neuroendocrine carcinoma. Five patients had *EGFR* mutations, 1 had a *KRAS* mutation, and 1 had an *ALK* fusion. Twenty-eight (66.7%) patients had more than 2 metastatic sites at the initiation of immunotherapy. Thirty (71.4%) patients received systemic therapies of less than the third-line setting prior to immunotherapy. Thirty-six (85.7%) patients received platinum-based therapies before the initiation of ICIs, 27 (64.3%) patients received radiotherapy before ICIs, and 2 (4.8%) patients received ATBs during ICI treatment. A significant difference was found between the R and NR cohorts in terms of the number of prior systemic regimens (*P* = 0.002). No significant difference was found in other patient characteristics between the R and NR cohorts.

### Gut microbiome diversity at baseline between responders and nonresponders to anti-PD-1 therapy

**Figure 2:**
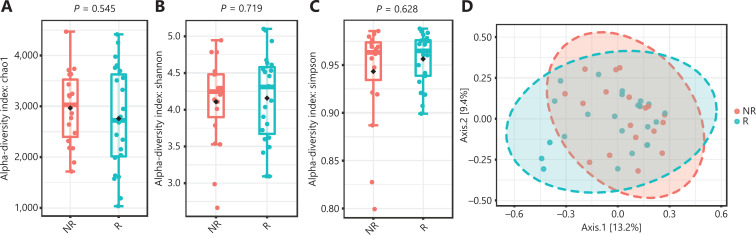
Gut microbiota diversity between the responder (R, *n* = 23) and nonresponder (NR, *n* = 19) groups at baseline. (A, B, C) Chao1 index, Shannon index, and Simpson index between the R and NR groups at the operational taxonomic unit level. Statistical analysis was performed using the Mann-Whitney test. *P* > 0.05 for all tests. (D) Principal coordinate analysis was based on the Bray-Curtis distance. *P* > 0.05 was determined by permutational multivariate analysis of variance.

Rarefaction curve analysis was performed and tended to reach a plateau, indicating that the sequencing depth was sufficient to estimate the total microbial diversity (**Supplementary Figure S2A and S2B**). The alpha diversity captures the richness and evenness of the OTU distribution in the community^33^. The Chao1 index, Shannon index, and Simpson index were then selected for the analysis of alpha diversity. There was no significant difference in the Chao1 index, Shannon index, or Simpson index at the OTU level between the R and NR groups at baseline (*P* = 0.545, *P* = 0.719, and *P* = 0.628, respectively; **Figure 2A, 2B, and 2C**). We further analyzed the beta diversity between the two groups, which reflects the similarity or difference between sample groups^33^. The beta diversity results were visualized by PCoA. PCoA based on the OTU profile showed no separation between the two groups (**Figure 2D**).

### Differences in the baseline microbiome compositions between the two groups

**Figure 3:**
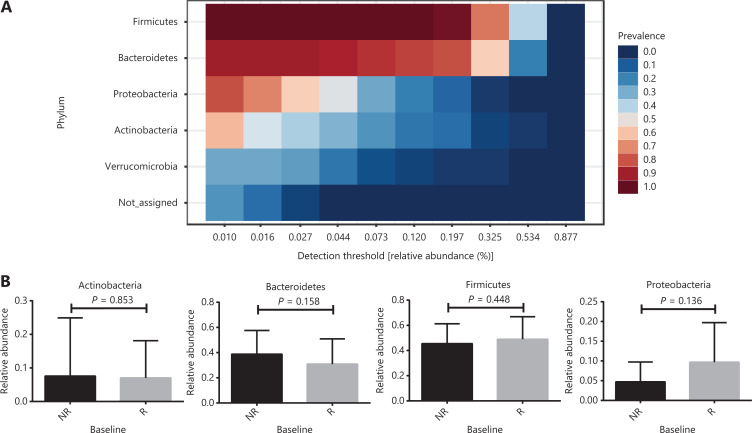
Gut microbial percentages at the phylum level in anti-PD-1 responding patients and nonresponding patients. (A) The core phyla microbiome in the current study. Bacterial phyla among samples prevalence above 20% and a relative abundance of greater than 0.01% are presented. (B) Comparison of the 4 main bacterial phyla between responders (R, *n* = 23) and nonresponders (NR, *n* = 19). Statistical analyses were performed using the Mann-Whitney test. *P* > 0.05 for all tests.

**Figure 4:**
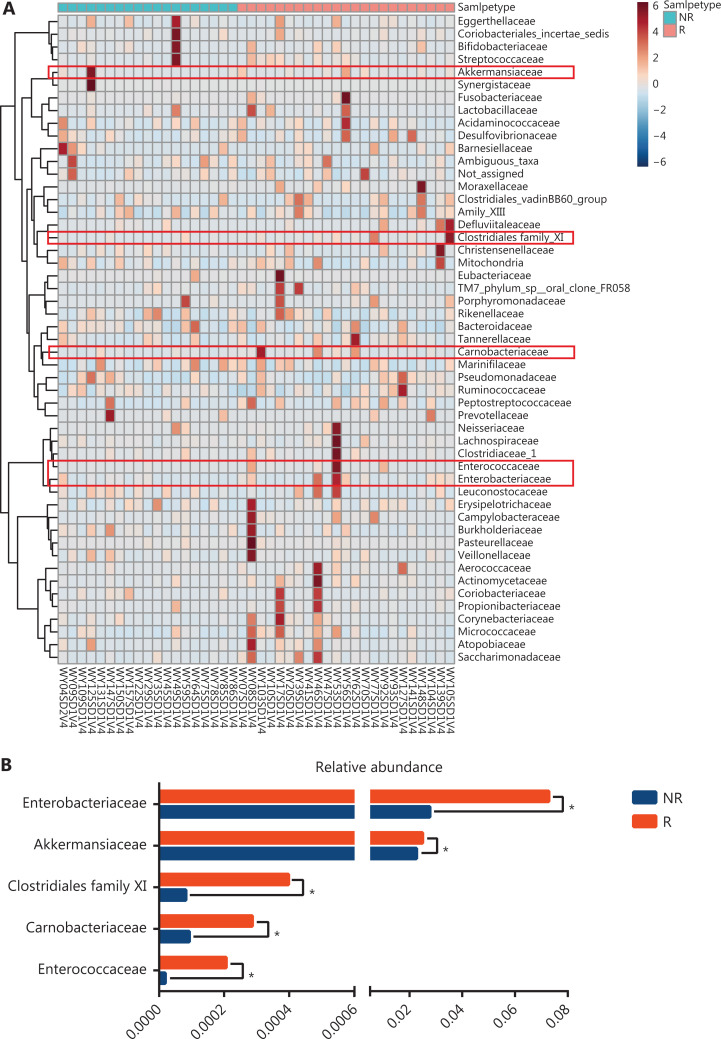
Comparison of the gut microbiota at the family level between responders (R, *n* = 23) and nonresponders (NR, *n* = 19). (A) Heat map visualization of gut microbiota at the family level prior to anti-PD-1 treatment. Columns represent individual patients clustered using hierarchical clustering with Euclidean distances. (B) Five bacterial families had significantly different abundances at baseline. Statistical analyses were performed using the Mann-Whitney test. *P < 0.05.

*Firmicutes*, *Bacteroidetes*, *Proteobacteria,* and *Actinobacteria* were the main microbiomes at the phylum level in the current study (**Figure 3A**). However, they were not significantly different between the R and NR groups (**Figure 3B**). We then compared the different compositions at the family level and identified 5 bacterial families that were more abundant in the R group than in the NR group (**Figure 4A, 4B, and Supplementary Table S1**). *Akkermansiaceae* and *Enterococcaceae* were enriched in the R group (*P* = 0.041 and *P* = 0.023, respectively). *Akkermansia* and *Enterococcus* are commensal genera in patients with NSCLC who benefit from anti-PD-1 therapy^17^. The bacterial genus *Granulicatella*, which represents the majority of the *Carnobacteriaceae* family, was significantly associated with superior clinical responses (*P* = 0.039, **Supplementary Figure S3 and Table S2**). Some studies also showed that the bacterial genera, *Granulicatella* and *Peptoniphilus*, as well as the bacterial family, *Enterobacteriaceae*, are found in the normal gut microbiota and are commensal microbes^34–36^.

### Association between baseline commensal microbes and PFS

**Figure 5:**
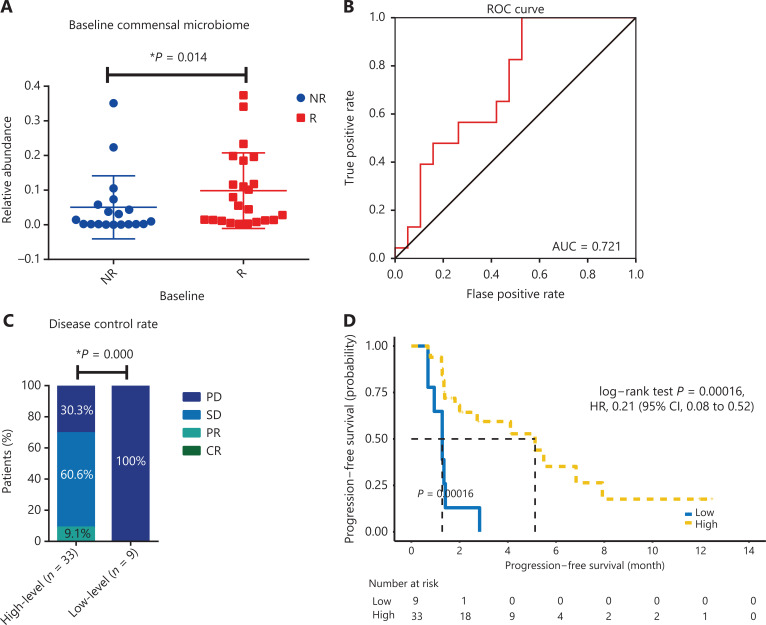
The association between the baseline commensal microbiome and patient clinical responses to anti–PD-1 therapy. (A) Comparison of the baseline commensal microbiome in responders versus nonresponders. Statistical analysis was performed using the Mann-Whitney test. *P < 0.05. (B) Receiver operating characteristic (ROC) curve analysis. The performance of the classification was evaluated using ROC curves. (C) Disease control rate in patients with high versus low levels of the baseline commensal microbiota. *P* values were calculated using the Fisher test. (D) Kaplan-Meier plot of progression-free survival with log-rank tests in patients with high versus low commensal microbiota levels.

Five bacterial families enriched in the R group were regarded as a commensal consortium, and the relative abundance of the commensal consortium stratified patients more accurately according to clinical responses (*P* = 0.014; **Figure 5A**). Receiver operating characteristic curve analysis was performed, and the cut-off abundances of the 5 bacterial families, as well as the commensal consortium, were calculated. The cut-off value of the commensal consortium was 0.003, which stratified patients with high levels of commensal microbes versus low levels of commensal microbes (**Figure 5B**). Next, we examined the impact of baseline commensal microbes on the response rate. None of the patients achieved a complete response (CR) in the current study. A high level of commensal microbes compared with the low level group was not different in terms of ORR, but significantly associated with a higher DCR (*P* = 0.000; **Figure 5C and Supplementary Table S3**). The median follow-up was 13.44 months [95% confidence interval (CI): 11.62–15.25]. The median PFS for all patients in this study was 2.82 months (95% CI: 0.41–5.24) and the median OS was 16.00 months (95% CI: 11.84–20.16). Baseline fecal commensal microbes were significantly associated with the patient PFS (*P* = 0.00016; **Figure 5D**); however, it did not influence the OS (*P* = 0.84; **Supplementary Figure S5**). Patients with a higher commensal bacterial abundance had a prolonged PFS. More precisely, the *Akkermansiaceae, Enterococcaceae, Enterobacteriaceae, Carnobacteriaceae,* and* Clostridiales Family XI* were all over-represented at diagnosis in patients with longer PFS (**Supplementary Figure S6A–6E**). The predictive capability of baseline commensal microbes was evaluated using Cox proportional hazards regression. Using univariate analysis, the mutation status of patients (*P* = 0.011), the number of prior systemic regimens (*P* = 0.000), and baseline fecal commensal microbes (*P* = 0.000) were all significantly associated with PFS. Moreover, using multivariate analysis, the baseline fecal commensal consortium and the number of prior systemic regimens were still significant factors affecting PFS (*P* = 0.003 and *P* = 0.006, respectively; **Table 2**).

**Table 2:** Univariate and multivariate analyses for progression-free survival in the cohort

Covariates	Comparisons	Univariate analysis	*P*	Multivariate analysis	
		Median PFS in months (95% CI)		*P*	HR (95% CI)
Age	≥65 *vs.* <65	5.13 (2.02–8.23) *vs.* 1.41 (0.03–2.80)	0.345		
Gender	Male *vs.* female	4.11 (0.47–7.45) *vs.* 1.81 (0.20–3.41)	0.626		
Histology	Squamous *vs.* adenocarcinoma	5.13 (2.19–8.07) *vs.* 1.81 (1.01–2.60)	0.119		
	Others *vs.* adenocarcinoma	1.28 (0.67–1.89) *vs.* 1.81 (1.01–2.60)	0.861		
Smoking status	Smoker *vs.* non-smoker	2.83 (0.00–6.05) *vs.* 2.73 (0.75–4.71)	0.994		
ECOG PS	0 *vs.* 2	5.49 (NA) *vs.* 2.73 (1.10–4.36)	0.547		
	1 *vs.* 2	4.11 (0.36–7.85) *vs.* 2.73 (1.10–4.36)	0.476		
Mutation status	*EGFR*/ *ALK* *vs.* others	1.35 (0.12–2.57)* vs.* 5.13 (1.64–8.61)	0.011*	0.701	0.74 (0.16–3.49)
Metastasis sites	<2* vs*. ≥2	5.49 (0.00–11.31) *vs.* 2.00 (0.71–3.30)	0.199		
Number of prior systemic regimens	<3 *vs.* ≥3	5.49 (3.64–7.34) *vs.* 1.28 (1.25–1.32)	0.000*	0.006*	0.15 (0.04–0.58)
Previous systemic therapy	Other systemic therapy *vs.* platinum-based therapy	6.83 (NA) *vs.* 2.83 (0.20–5.45)	0.987		
Prior radiotherapy	Yes *vs.* no	5.13 (1.63–8.62) *vs.* 1.38 (1.26–1.50)	0.276		
Usage of ATB	Yes *vs.* no	0.69 (NA) *vs.* 2.83 (0.40–5.25)	0.671		
Commensal microbiome	High *vs.* low	5.13 (1.52–8.73) *vs.* 1.28 (0.85–1.71)	0.000*	0.003*	0.17 (0.05–0.55)

*P < 0.05 was considered significant.

### The gut microbiota composition is similar during anti-PD-1 treatment.

**Figure 6:**
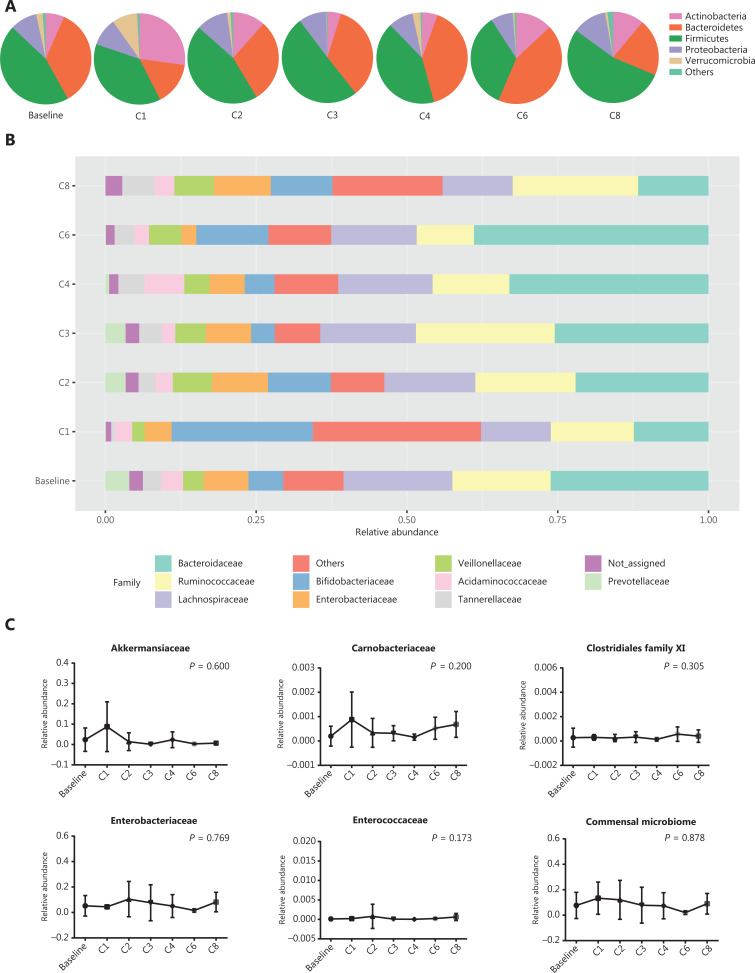
Gut microbiota profiles during anti-PD-1 treatment. (A) Main bacterial phylum percentages over the course of anti-PD-1 treatment. (B) Main bacterial family percentages over the course of anti-PD-1 treatment. (C) The percentages of commensal bacterial families over the course of anti-PD-1 treatment. Statistical analysis was performed using the Kruskal-Wallis test. *P* > 0.05 for all tests.

To better characterize the abundance of gut commensal bacteria, time-serial fecal samples were collected during anti-PD-1 therapy. Diversities calculated by both the Shannon and Simpson indices were not altered by anti-PD-1 therapy (*P* = 0.434 and *P* = 0.807, respectively; **Supplementary Figure S4A and S4B**). Four main bacterial phyla, namely, *Actinobacteria*, *Bacteroidetes*, *Firmicutes,* and *Proteobacteria*, remained stable from baseline to different time points (Kruskal-Wallis test; *P* > 0.05; **Figure 6A**). As shown in **Figure 6B**, the bacterial percentages at the family level were not significantly altered during the treatment (Kruskal-Wallis test; *P* > 0.05). Moreover, the percentages of 5 bacterial families abundant in the R group at baseline, as well as the commensal microbiome, slightly fluctuated over the course of anti-PD-1 treatment (Kruskal-Wallis test; *P* > 0.05; **Figure 6C**). Combined analyses of the data showed that the gut microbiome composition was not significantly modified by anti-PD-1 therapy in patients with advanced thoracic carcinoma.

## Discussion

This study showed that compositional differences in the baseline gut microbiome were associated with anti-PD-1 therapeutic responses in patients with thoracic neoplasms. The relative abundances of 5 bacterial families (*Akkermansiaceae*, *Enterococcaceae*, *Carnobacteriaceae*, *Enterobacteriaceae,* and *Clostridiales Family XI*) were significantly different between the R and NR groups, and they were all over-represented in the R group. The high level of the commensal microbiome group also exhibited an increased DCR and longer PFS. In addition, the relative abundance of this baseline commensal consortium was an independent risk factor for checkpoint blockade therapy responses.

Routy et al.^17^ reported that *Akkermansia* and *Enterococcus* were commensal bacterial genera, and were significantly associated with clinical responses in NSCLC. They also reported that *Akkermansia* was enriched in patients with a longer PFS^17^. Our results confirmed these conclusions at the family level. Moreover, our study showed that the *Enterobacteriaceae*, *Carnobacteriaceae,* and* Clostridiales Family XI* bacterial families were also associated with favorable responses to anti-PD-1 treatment. Although the immune system of the host is activated by infection, *Enterobacteriaceae* is required by intestinal epithelial cells (IECs) to clear the pathogen^36^. In mouse models of *Citrobacter rodentium* infection, inflammation changes the metabolic landscape of IECs and leads to the colonization of anaerobes^37^. The growth of the *Enterobacteriaceae* family switches the metabolism and oxygen availability of IECs, which might cooperate with the innate immune response of the host^38^. In the present study, the main genus detected in the *Carnobacteriaceae* family was *Granulicatella*. Until now, there has been no report of the beneficial roles of gut *Granulicatella* in patients with thoracic neoplasms during anti-PD-1 therapy. A previous study of HIV-infected individuals showed that *Granulicatella* was a commensal microbe in the respiratory tract^39^. Katagiri et al.^40^ showed that the *Carnobacteriaceae* family and *Granulicatella* genus, which were over-represented in the gut microbiome after rehabilitation for dysphagia, affected the systemic health of stroke survivors. Moreover, in the current study, the richness of the *Carnobacteriaceae* bacterial family was relatively increased in the R group, indicating that *Carnobacteriaceae* in the gut benefited anti-PD-1 therapeutic responses. The *Clostridiales family XI* family was also significantly enriched in the R group. Many members of the *Clostridiales family XI*, including *Peptoniphilus*, have been shown to utilize peptone as the major metabolic product^41^. This genus colonizes the normal gut and upper respiratory tract of humans^35^. Many species of this genus can act as opportunistic pathogens in immunocompromised patients and are associated with polymicrobial infections^42–44^. Because this genus contains not only pathogenic species but also probiotic species, future studies will be needed to verify the relationship between anti-PD-1 therapy and this bacterial genus.

Among clinical characteristics, the present study showed that the number of prior systemic regimens was associated with responses to anti-PD-1 therapy. Using multivariate analyses, patients receiving third-line or the above systemic treatments prior to ICIs had a shorter PFS. The result was mainly due to worse performance status and a relatively larger tumor burden in the heavily treated patients. The number of prior regimens was also associated with decreased OS in another cohort of patients with NSCLC receiving ICI therapy^45^. Previous studies have shown that gut microbiota are not modified by ICI treatment in patients with NSCLC and metastatic melanoma^28,46^. Our study also found that the gut microbiota composition, especially commensal bacteria, remained stable in serial stool samples. This suggested that a higher level of the commensal microbiome at baseline provided long-term benefits for patients receiving ICI therapy.

A diverse array of commensal bacteria that reside in the gastrointestinal tract are important entry sites against invasion from pathogens, and the intestinal immune system is devoted to protecting against infections and other diseases^47,48^. Evidence has emerged that there is a strong correlation between commensal bacteria and clinical responses to immunotherapy. Matson et al.^27^ showed that the ratio of beneficial bacteria to “non-beneficial” bacteria could be a predictor of clinical responses to checkpoint blockade therapy in patients with metastatic melanoma. In mouse models of melanoma, the oral administration of commensal *Bifidobacterium* improved dendritic cell function and tumor-specific CD8^+^ T cell responses, promoting anti-tumor immunity involving checkpoint blockade^49^. Furthermore, 11 low abundance commensal strains of human gut microbiota induced the accumulation of interferon-γ-producing CD8 T cells and simultaneously enhanced ICI therapy^50^. Importantly, our study also showed that 5 bacterial families that were abundant in the responders might act as a commensal consortium that more accurately stratified patients according to clinical responses.

There were some limitations in our study. First, the number of patients enrolled in the present study was relatively small, and the findings require validation in another larger, independent cohort. In addition, due to the limited resolution of 16S sequencing, long-read sequencing or shotgun sequencing are needed to distinguish between related bacterial families. Moreover, owing to the issues mentioned above, we failed to detect bacteria with a negative impact on ICI therapy at baseline, which might also be important in clinical responses^27^.

## Conclusions

Our findings provided an example of gut-resident commensal microbiota that were associated with a favorable response to anti-PD-1 therapy in patients with thoracic neoplasms. Importantly, when a high level of the commensal consortium was present in the pretreatment feces, patients had a better prognosis.
