# Supplementary material for: The commensal consortium of the gut microbiome is associated with favorable responses to anti-programmed death protein 1 (PD-1) therapy in thoracic neoplasms
Source: Cancer Biol Med. 2021 May 7;18(4):1040–52. doi: 10.20892/j.issn.2095-3941.2020.0450 (PMC8610161; doi:10.20892/j.issn.2095-3941.2020.0450)
Supplement: Supplementary file 1 [file cbm-18-1040-s001.pdf]

Supplementary materials

PATIENT ID	BASELINE	C1	C2	C3	C4	C6	C8
WY04	●	●					
WY07	●		●		●	●	
WY08	●						
WY09	●		●	●	●		
WY10	●		●		●	●	●
WY17	●			●		●	
WY20	●						
WY22	●			●			
WY29	●		●				
WY35	●						
WY39	●		●		●	●	●
WY41	●			●			
WY45	●			●			
WY46	●		●		●	●	●
WY47	●		●		●		
WY49	●						
WY53	●		●				
WY56	●		●		●		
WY59	●		●				
WY62	●		●				
WY64	●		●				
WY70	●						
WY75	●		●				
WY77	●		●		●		
WY78	●						
WY85	●						
WY86	●						
WY92	●		●	●			
WY93	●		●				
WY103	●		●				
WY104	●						
WY105	●		●				
WY109	●		●				
WY125	●		●				
WY127	●						
WY131	●		●				
WY139	●						
WY141	●						
WY147	●						
WY148	●						
WY150	●	●					
WY157	●						

Figure S1 A summary of each patient's sample time points.

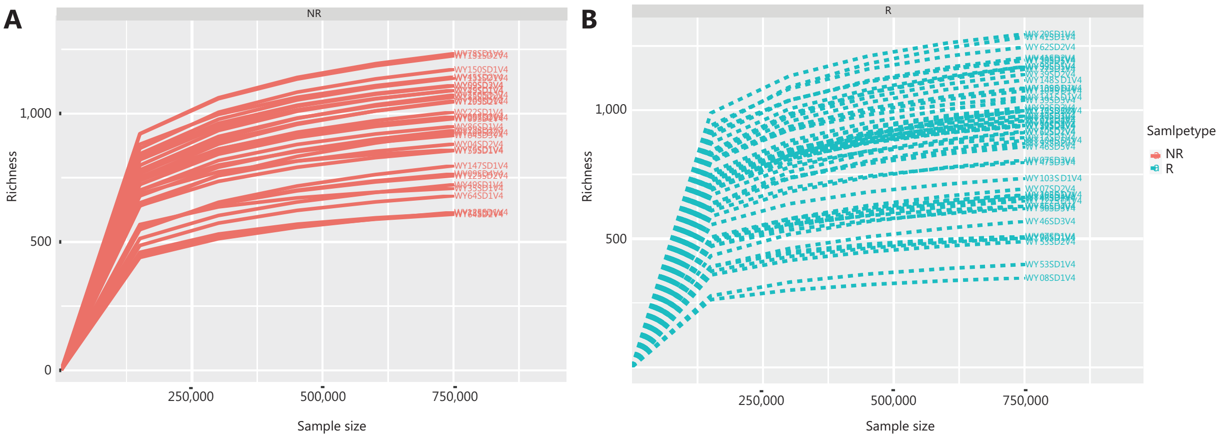

Figure S2 Rarefaction curves of fecal samples using 16S ribosomal RNA gene sequencing from the nonresponder (A) and responder groups (B).

A

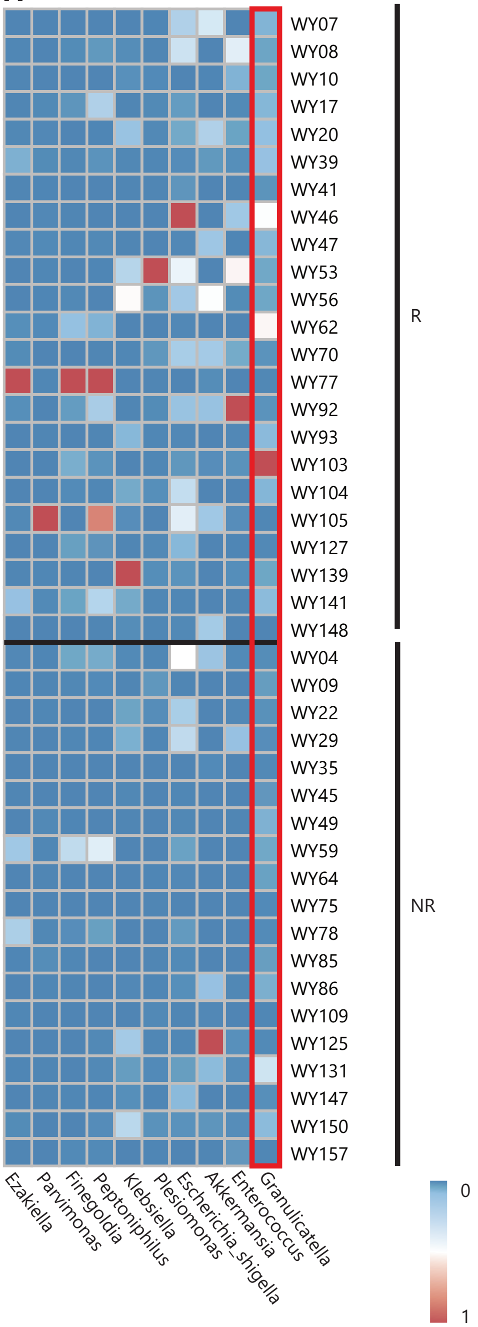

B

Family level	Genus level
Carnobacteriaceae	Granulicatella
Enterococcaceae	Enterococcus
Akkermansiaceae	Akkermansia
Enterobacteriaceae	Escherichia_shigella
	Plesiomonas
	Klebsiella
Clostridiales family XI	Peptoniphilus
	Finegoldia
	Parvimonas
	Ezakiella

C

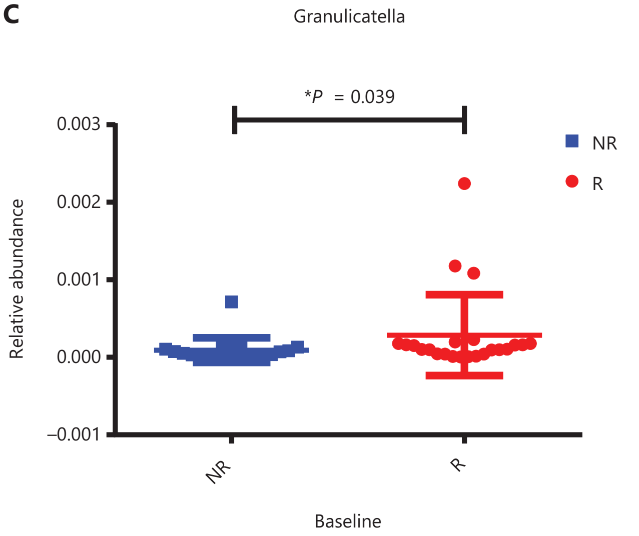

Figure S3 Comparison of 5 bacterial families with different abundances at the genus level. (A) Heat map of the dominant gut microbial genera belonging to the 5 bacterial families. (B) Description of the commensal microbiome summarized at the family and genus levels. (C) Comparison of the *Granulicatella* bacterial genus between responders (n = 23) and nonresponders (n = 19). Statistical analysis was performed using the Mann-Whitney test. *P < 0.05.

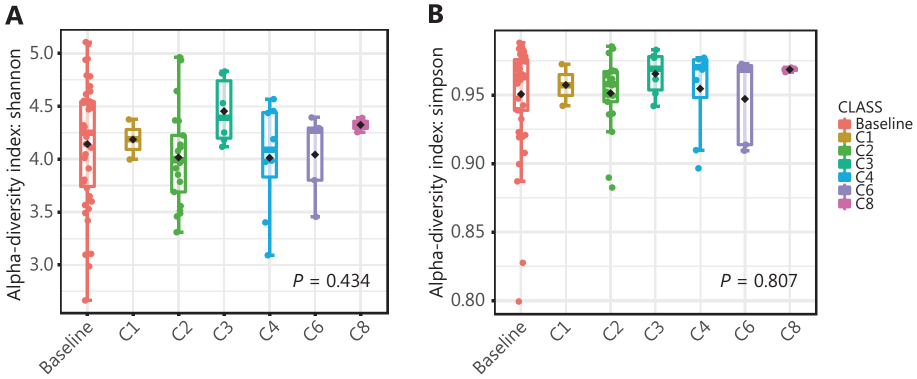

Figure S4 The diversity of the gut microbiome during anti-PD-1 treatment. Dynamics of the Shannon index (A) and Simpson index (B) in longitudinal stool samples. Statistical analysis was performed using the Kruskal-Wallis test. *P* > 0.05 for all tests.

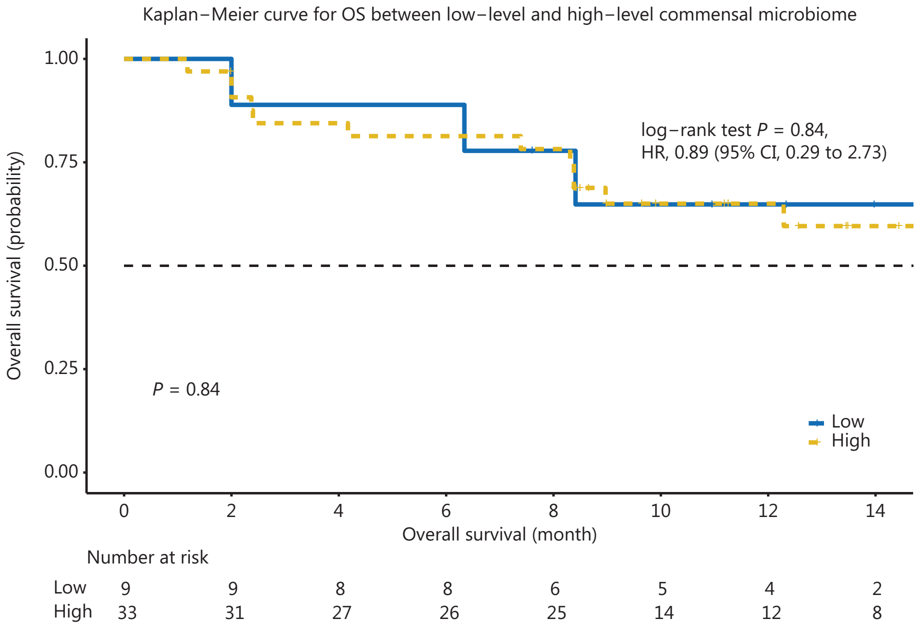

Figure S5 Kaplan-Meier plot of overall survival with log-rank tests in patients treated with immune checkpoint inhibitors, stratified by high versus low levels of the baseline commensal microbes.

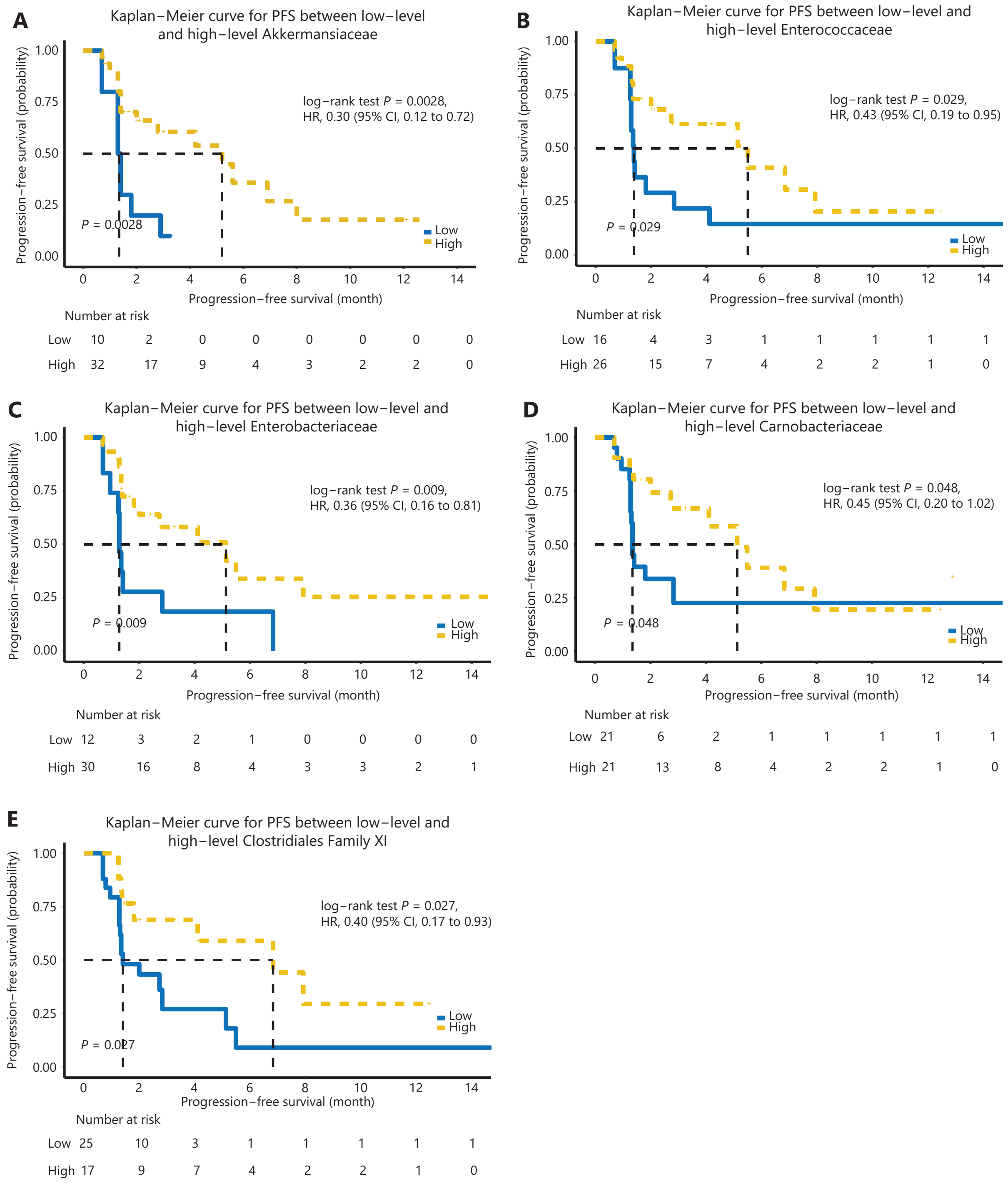

Figure S6 Kaplan-Meier plot of progression-free survival using log-rank tests in patients with high versus low levels of *Akkermansiaceae* (A), *Enterococcaceae* (B), *Enterobacteriaceae* (C), *Carnobacteriaceae* (D), and *Clostridiales Family XI* (E).
